# Deterministic Terahertz Wave Control in Scattering Media

**DOI:** 10.1021/acsphotonics.2c00061

**Published:** 2022-07-19

**Authors:** Vivek Kumar, Vittorio Cecconi, Luke Peters, Jacopo Bertolotti, Alessia Pasquazi, Juan Sebastian Totero Gongora, Marco Peccianti

**Affiliations:** † Emergent Photonics Lab (EPic), Department of Physics and Astronomy, University of Sussex, Brighton BN1 9QH, U.K.; ‡ Department of Physics and Astronomy, University of Exeter, Exeter, Devon EX4 4QL, U.K.

**Keywords:** THz imaging, scattering media, THz wave control, spatiotemporal focusing, coherent transfer matrix

## Abstract

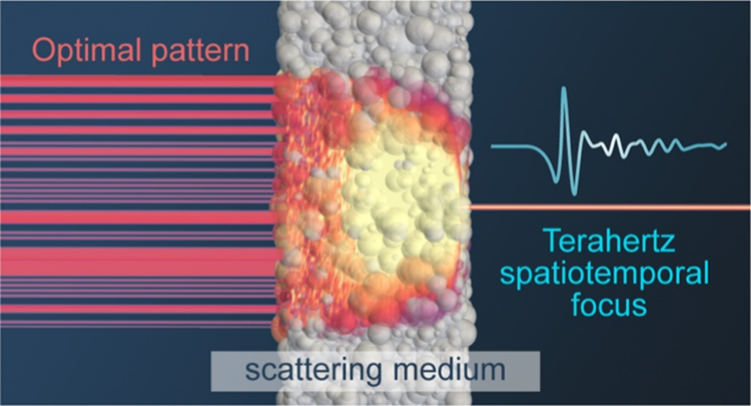

Scattering-assisted synthesis of broadband optical pulses is recognized to have a cross-disciplinary fundamental and application importance. Achieving full-waveform synthesis generally requires means for assessing the instantaneous electric field, i.e., the absolute electromagnetic phase. These are generally not accessible to established methodologies for scattering-assisted pulse envelope and phase shaping. The lack of field sensitivity also results in complex indirect approaches to evaluate the scattering space–time properties. The terahertz frequency domain potentially offers some distinctive new possibilities, thanks to the availability of methods to perform absolute measurements of the scattered electric field, as opposed to optical intensity-based diagnostics. An interesting conceptual question is whether this additional degree of freedom can lead to different types of methodologies toward wave shaping and direct field-waveform control. In this work, we theoretically investigate a deterministic scheme to achieve broadband, spatiotemporal waveform control of terahertz fields mediated by a scattering medium. Direct field access via time-domain spectroscopy enables a process in which the field and scattering matrix of the medium are assessed with minimal experimental efforts. Then, illumination conditions for an arbitrary targeted output field waveform are deterministically retrieved through numerical inversion. In addition, complete field knowledge enables reconstructing field distributions with complex phase profiles, as in the case of phase-only masks and optical vortices, a significantly challenging task for traditional implementations at optical frequencies based on intensity measurements aided with interferometric techniques.

## Introduction

The propagation of waves in a scattering medium results in complex space–time interference patterns, i.e., in a complex time- and position-dependent response at the output.^1,2^ These phenomena are ubiquitous features in the physics of random wave propagation and significantly impact applications in several domains ranging from electromagnetic to acoustic, mechanical, and matter waves.^2,3^ For instance, in optical imaging, random propagation of light rapidly reduces the image fidelity in deep biological tissue characterization.^4^ As such, the performance of state-of-the-art microscopes is traditionally affected by the ineliminable dynamical turbidity in the samples.^5,6^

Although light scattering is usually considered an impediment, it is not necessarily accompanied by an irreversible loss of information.^7^ By leveraging this principle, researchers have recently developed a broad range of wavefront-shaping techniques to control complex light propagation through a scattering medium.^8,9^ The basic principle is to spatially modulate the wave impinging onto the medium to harness the scattering-induced amplitude and phase distortions. Recently, various approaches toward optical wavefront compensation based on feedback,^10^ guide stars,^11^ and memory effect^12^ have been demonstrated in different disciplines. These methodologies have enabled the manipulation of scattered waves for refocusing and imaging applications. Although approaches based on the iterative optimization of the scattered field rely on technically simple implementations, they fundamentally operate without direct knowledge of the scattering medium. As such, a specific optimization process provides little clues for a different one, and convergence is usually established solely by the inability to reduce an error function further. Deterministic approaches overcome this limitation. They rely on the knowledge provided by measuring the optical transfer matrix of the medium.^13,14^ Deterministic methods first measure the scattered light field corresponding to different sets of amplitude^15,16^ or phase^17,18^ illumination patterns (preferably forming an orthogonal set). The measurements are then combined to achieve a single-step illumination retrieval for a desired optimized wavefront through numerical inversion.

### Challenges of Field-Wave Synthesis Using Random Media

Within the process of exploiting random media for space–time wave synthesis, one can argue that the knowledge of the transmission matrix is insufficient. While the transmission matrix components can be retrieved via complex spectral interferometry approaches, a full spatiotemporal synthesis requires prior knowledge of the source. If the absolute phase profile of the source pulse is not known, its effect on the scattered field is also unknown.

Interestingly, when the detection can resolve the instantaneous field dynamics for a large set of spatially modulated fields, one can trivially target a new scattered waveform as a simple combination of the field scattered by different illuminations without directly referring to the scattering matrix. Indeed, powerful wave-synthesis approaches in optics do not generally rely upon absolute phase knowledge.^18^ This rationale, for example, is one of the critical accelerating factors for optical frequency combs technology.^19^ Conversely, persuasive, popular nonlinear pulse diagnostics (e.g., FROG or SPIDER) do not provide access to the instantaneous field.^20−22^

### Full Time-Domain Field Approach

The ability to perform a complete time-domain detection brings a conceptual difference: by introducing a sparse-light modulation (as in the practice of random media functionalization) for each spatially orthogonal illumination *p_i_*, one can detect the corresponding space–time waveform *E_i_*^+^ (*x*_o_,*t*). These independently transmitted waveforms can, in principle, be used to decompose any desired space–time waveform *E*_T_^+^ (*x*_o_, *t*) at the output as a linear superposition
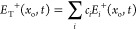
1where *c*_*i*_ are complex-valued expansion coefficients. Once one determines the set of *c*_*i*_, experimentally achievable, this can provide access to the spectrum of available waveforms. While this approach does not require specific access to the source waveform, the latter is, however, trivially accessible via time-sensitive detection and would grant access to the scattering matrix.

In this context, terahertz time-domain spectroscopy (THz-TDS) is a mature and established technique capable of fully resolving the electric-field oscillations in a broadband pulse,^23^ i.e., providing full knowledge of the complex spectral field.^24^ The scientific question is whether THz-TDS can be exploited to develop a deterministic approach to waveform synthesis (closely related to time-reversal methods,^25−27^ ultrasound, or radiofrequency approaches^28,29^). The idea is to extract sufficient information to obtain access to any scattering-allowed output field. The large relative THz bandwidth available in TDS embodiments (normally exceeding a decade) allows easily spanning a wide range of single and multiple scattering regimes for a given sample.^30,31^ On the practical side, the relatively large wavelength of THz waves (spanning from roughly 30 μm to 3 mm) suggests that the typical subwavelength scales of scattering phenomena are significantly more accessible in experimental platforms when compared to optical embodiments.^32−34^

A general downside in implementing THz wavefront control methods is, however, the limited availability of wavefront-shaping devices.^35,36^ In addition, the use of diffraction-limited systems at long wavelengths (which fixes the pattern resolution^37,38^) is undesirable because the experimental setting usually does not involve samples several orders of magnitude larger than the wavelength, trivial conditions in optics. This results in a relatively small number of modes that can be independently excited in a scattering structure with far-field illumination.^39^ Very recently, the nonlinear conversion of structured optical beams has emerged as a promising approach toward deeply subwavelength spatial light modulation (SLM) of THz waves.^40,41^ The combination of nonlinear THz pattern generation and time-resolved field detection, in particular, has enabled the development of hyperspectral THz imaging with deep subwavelength imaging resolution.^42^ In essence, placing an object in the near-field of a nonlinear optical-to-THz converter makes it possible to produce terahertz illumination patterns with fine spatial features approaching the optical (i.e., the pump) diffraction limit.

In this work, we explore this framework in connection with scattering-assisted waveform synthesis, introducing the field equivalent of the traditional spatiotemporal focusing and image retrieval. We explore scenarios extremely challenging in optics, which include retrieval of field distributions with complex phase profiles, such as phase-only masks and optical vortices. In our approach, we expand the complex-valued, coherent transfer matrix of the scattering medium using an orthogonal Walsh–Hadamard decomposition of near-field THz illumination.^43^ We leverage this knowledge to perform a direct single-step inversion using a constraint least-square optimization approach compatible with realistic experimental conditions.^44^

## Methods

### Model Definition and Simulation Setup

We define the input/output field relation in terms of an impulse response *T*_*x*_ (*x*_o_, *x*′, *t*, *t′*),^45^ as
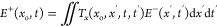
2where *E*^–^ and *E*^+^ denote the spatiotemporal electric-field distribution just before and after the scattering medium. To lighten the notation, we define *x*_o_ and *x*′ as the one-dimensional representation of the input and output planes, respectively. In the frequency domain ω, eq 2 reads
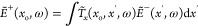
3where *Ẽ*^+^ (*x*_o_, ω) and *Ẽ*^–^ (*x*′, ω) are the time-Fourier transforms of the input and output fields.

Following standard approaches, we rewrite eq 3 in a discrete scalar transfer matrix formalism, where the response of the scattering medium for each incident frequency is described by an *M* × *N* field-based, random transmission matrix *T_mn_* ∈ ^*M*×*N*^.^46^ We divide the output and input planes into *M* and *N* spatial independent segments (corresponding, e.g., to the physical pixels on the input wavefront-shaping and output imaging devices) and *K* spectral modes, a representation that is well-suited for experiments. In the discrete coordinates, the transfer matrix is an *N* × *M* × *K* three-dimensional matrix defined as *T_mnk_* = *T_mn_*(ω_*k*_). For a given *k*th frequency ω_*k*_, the relationship between the THz fields *E*_*m*_^+^ (ω_*k*_) and *E*_*n*_^–^ (ω_*k*_) at the *n*th input and *m*th output pixels reads
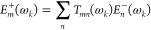
4where we considered a coherent transfer matrix defined as
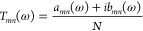
5with *a*_*mn*_(ω) and *b*_*mn*_(ω) random Gaussian variables with zero mean, and standard deviation σ = 1/2.^47^ The 1/*N* normalization factor ensures that, on average, the power transmitted by the TM is roughly half of the incident power. To introduce a controllable spectral correlation in the new model, we applied a Gaussian filter of width Δω_c_ to the real and imaginary part of the transfer matrix.^46,48−50^ As illustrated in Figure S1, we verified that the introduction of a spectral correlation does not affect the statistical properties of the transfer matrix elements for each individual frequency. The spectral correlation is directly associated with the sample properties and inversely proportional to the Thouless time (corresponding to the average confinement time of the field in the medium).^49,50^ In the presence of broadband illumination, the spectral correlation bandwidth Δω_c_ is of critical importance, as it determines the total number of accessible spectral modes within the illumination bandwidth.^9,39,51^ In our case, we convolve a white-noise distribution with a Gaussian filter with a standard deviation of Δ*ν*_c_ = Δω_c_/2π = 150 GHz along the frequency axis to impose a desired spectral correlation in the transfer matrix elements. Further details on our particular choice of parameters are included in Supporting Information Note 1.

**Figure 1:**
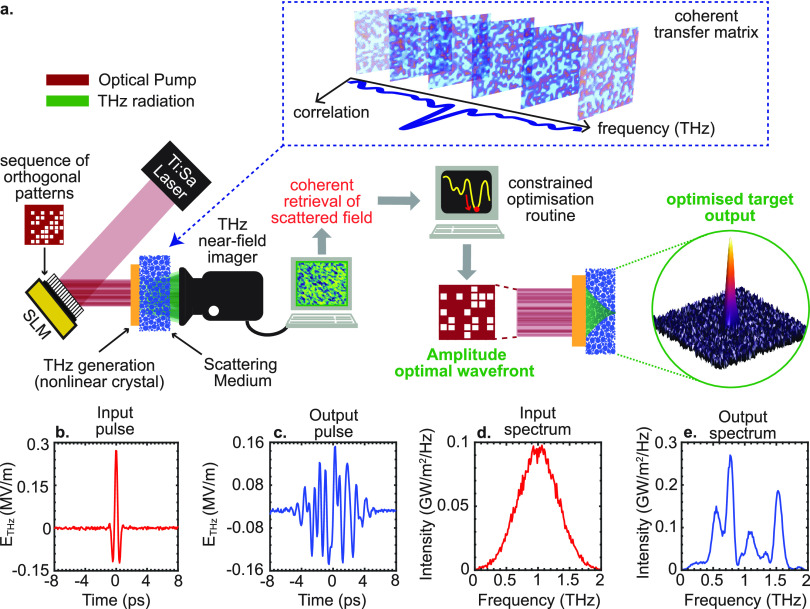
Schematic of experimental-driven methodology (a) Conceptual overview of methodology, including the nonlinear conversion of optical patterns to THz structural waves and the retrieval of transmission properties of the scattering medium defined in terms of a coherent transfer matrix. The full knowledge of the coherent transfer matrix retrieved using an orthogonal set of patterns can be used to achieve scattering-assisted focusing at the output of the scattering medium. (b) Input THz pulse electric-field profile. (c) Scattered THz pulse collected at a generic *m*th output pixel. (d) Intensity spectral density of the input THz field. (e) Intensity spectral density of the scattered THz pulse as collected at a generic *m*th pixel. In our simulations, we considered a 1 nJ THz pulse of duration 250 fs at the input with 40 dB SNR per pixel. The 6.4 × 6.4 mm^2^ sample illumination area is spatially sampled at 200 μm resolution, corresponding to a number of pixels of 32 × 32.

Figure 1a provides a conceptual overview of the THz-TDS experimental configuration we referenced in our modeling. An optical spatial light modulator (SLM) impressed a desired spatial pattern on an ultrafast optical field (λ = 800 nm). The optical pattern is converted to a THz structured field via a nonlinear crystal, as discussed in ref (41). Without loss of generality, we assume a quadratic χ^(2)^ optical rectification process (e.g., ZnTe) that converts the optical intensity wavefront *I*^optical^ (*x*′) to a THz wavefront *E*^THz^ (*x*, *t*) as follows
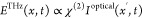
6where χ^(2)^ is the second-order susceptibility of the nonlinear crystal. With this position, the THz field impinging on the scattering medium is defined in the frequency domain as *Ẽ*^–^ (*x*′, ω) = *I*^optical^ (*x*′) *f*(ω), where *f*(ω) is the spectrum of the THz pulse. Our scheme requires controlling the optical intensity distribution impinging on the nonlinear crystal. This could be easily achieved in experiments through an amplitude SLM (e.g., a Digital Micromirror Device or DMD)^41,52^ or phase-only SLMs combined with interferometric techniques.^53^ The THz pattern impinges upon the scattering medium and produces a complex, time-dependent interference pattern at the output. Finally, a TDS image of the scattered THz wave is collected through a parallel, near-field imaging scheme based on electrooptical sampling.^54^ To assess the robustness of our numerical approach to experimental noise, we performed the theoretical analysis in this manuscript by assuming a 40 dB signal-to-noise ratio (SNR) per pixel at the detection. With this assumption, the THz pulse contains a white-noise term that is compatible with the experimental conditions. Such a relative low noise can be easily achieved in experiments based on electrooptic detection using balanced detectors of the THz field-induced birefringence in a nonlinear crystal.^55^ In Figure 1b–e, we show an illustrative THz transmitted field as a function of the spatial and spectral coordinates as obtained for plane wave illumination and detected in a single pixel at the output. The temporal profile of the pulse is significantly broadened (Figure 1c), and the peak field is attenuated. When moving to the spectral domain (Figure 1e), the transmitted THz field is characterized by a random modulation of its spectrum as a consequence of interference and dispersion effects induced by multiple scattering.

**Figure 2:**
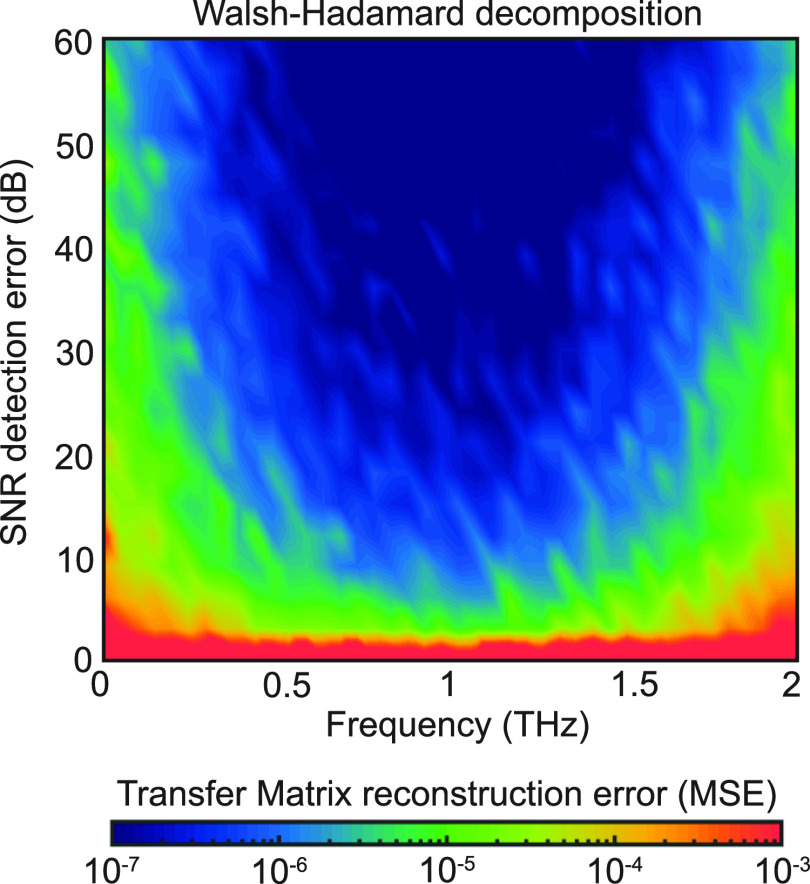
Transfer Matrix reconstruction. Mean squared error (MSE) of the coherent transfer matrix elements as reconstructed through a Walsh–Hadamard decomposition. The input and output planes are divided into 16 × 16 pixels, corresponding to a scattering matrix composed of 256 × 256 entries.

In close analogy with the traditional experimental approaches, we simulated the reconstruction of the coherent transfer matrix by computing the TDS output images corresponding to a predefined set of illumination patterns. In our simulations, we employed a Walsh–Hadamard decomposition scheme, i.e., we determined the full-wave responses corresponding to each column of an *N* × *N* Walsh–Hadamard matrix. We then extracted the frequency-dependent elements of the coherent transfer matrix through a linear inversion of the Walsh–Hadamard response (Figure 2). The detailed reconstruction method is described in Supporting Information Note 2.

The identification of an optimized optical spatial pattern *I*_opt_ (*x*′) that produces a given field profile of interest *Z̃*_target_ (*x*_o_, ω) carries a few significant challenges. First, as can be easily evinced from eq 6, in optical rectification, the THz field phase cannot be controlled through the incident optical phase, i.e., we can only control the THz amplitude distribution by varying the intensity distribution of the optical pump. Second, the spatial distribution of the optical intensity pattern is bound to be the same for all of the different frequencies carried by the THz pulse. Due to these two constraints, we cannot invert the coherent transfer matrix directly as the solution pattern is likely a frequency-dependent amplitude and phase distribution. On the contrary, we must identify a single, amplitude-only field distribution that best approximates the desired field distribution at the output. It is essential to stress that this is a post-measurement process, as opposed to the case of typical optimization techniques relying on feedback loops between illumination and measurement.

To this end, we cast our inversion problem in terms of a constraint least-square minimization of the following fitness function
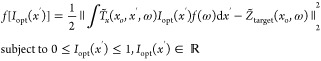
7

where ∥···∥_2_ is the Euclidean norm. In the discrete coordinate system, eq 7 reads
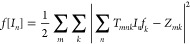
8where *T*_*mnk*_ = (*x*_*m*_, *x*_*n*_^*′*^, ω_*k*_), *I*_*n*_ = *I*_opt_ (*x*_*n*_^′^), *f*_*k*_ = *f*(ω_*k*_), and *Z*_*mk*_ = *Z̃*_target_ (*x*_*m*_, ω_*k*_), and where we replaced the Euclidean norm with the Frobenius norm. The constrained convex optimization problem defined in eqs 7 and 8 can be solved using various techniques. We use the Trust-Region-Reflective algorithm, a well-established method capable of rapidly solving relatively large-scale problems with low memory requirements.^56^ The ability to optimize the full-field properties of the transmitted field is a distinctive feature of this approach; eqs 7 and 8 are, indeed, an absolute phase-sensitive optimization, corresponding to a field-driven best fit rather than an intensity-driven fit.

## Results and Discussion

### Spatiotemporal Focusing of THz Waves through a Scattering Medium

**Figure 3:**
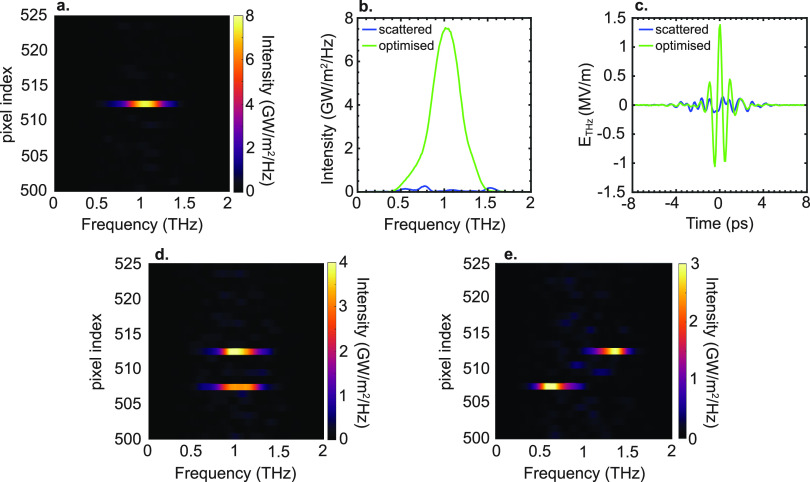
Spatiotemporal focusing of THz field: (a) optimized intensity spectral density distribution showing the focus spot in THz band. (b) Comparison between intensity spectral density profiles of the perturbed THz spectrum (blue) and optimized spectrum (green) at the *m*th pixel. (c) THz pulse profile of scattered field (blue) and optimized field (green) from *the m*th pixel of the output plane. (d) Intensity spectral density distribution of the output THz field showing two simultaneous focus spots at *m*th pixel and *m*^′^th pixel. (e) Intensity spectral density distribution of optimized THz field for two simultaneous focus spots at *m*th and *m*′th pixel with two different spectra centered around 0.7 and 1.3 THz. SNR per pixel: 40 dB. The 6.4 × 6.4 mm^2^ sample illumination area is spatially sampled at 200 μm resolution, corresponding to a number of pixels of 32 × 32.

Our first objective is to invert the coherent transfer matrix to obtain a spatiotemporal localized focus spot at the output of the scattering medium, a classical state-of-the-art scenario. Such a task has been explored in the optical and infrared domain both for monochromatic^15,52^ and ultrafast pulses,^25,50,57−59^ but never tackled for THz fields. In our approach, the realization of a spatiotemporal focus corresponds to imposing the following target field profile in eq 7
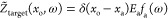
9where *x*_a_ is the desired focus position, and *E*_a_*f*_a_ (ω) is the spectrum of the incident THz pulse. Equation 9 targets an output field localized in one spatial point with the same spectral profile as the incident pulse. The results are shown in Figure 3a–c and effectively predict the formation of a sharp focus at the output. Quite remarkably, our amplitude-only optimized wavefront yields a spectral intensity enhancement η (defined as the ratio between the optimized and incident intensity spectral density at a specific frequency) of 78.43 (at 1 THz) at the focus spot (Figure 3b). The field peak (Figure 3c) is enhanced by a factor of 5.10, whereas the field-temporal standard deviation (the transient duration) is compressed by a factor of 4 with respect to the unoptimized case. It is worth stressing that, by observing Figure 3c, not just the pulse is recompressed. As expected from a full-field function reconstruction, the field dynamics are reconstructed locally, similar to Figure 1b. As illustrated in Figure S3, the performance of the optimal pattern is virtually identical to those obtained with standard iterative optimization techniques.^60,61^

Our approach is easily extendable to more challenging conditions, including the formation of separate spatial foci with different spectral profiles. To this end, we generalized eq 7 to the case of two foci by imposing
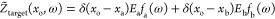
10where *x*_a_ and *x*_b_ correspond to two different focus locations, and *E*_a_*f*_a_(ω) and *E*_b_*f*_b_(ω) denote two distinct spectral profiles, respectively. Our results are shown in Figure 3d,e. When considering identical spectral profiles at the output (Figure 3d, *f*_a_ (ω) = *f*_b_ (ω)), we achieved the THz intensity spectral densities of 4.087 GW/m^2^/Hz (η = 43.10) and 3.17 GW/m^2^/Hz (η = 33.43) at 1 THz and THz peak field enhancements of 3.64 and 3.73, respectively. When considering two different spectral profiles, centered at around 1.3 and 0.7 THz, respectively (Figure 3e), the two foci exhibit intensity spectral densities of 2.74 GW/m^2^/Hz (η = 28.94) and 2.43 GW/m^2^/Hz (η = 25.63), respectively. For the sake of clarity, we stress here that the two spectral profiles included in eq 10 are different from the incident pulse spectrum.

### Deterministic Coherent Control without Previous Knowledge of the Source

The loss function defined in eq 7 targets the spatial distribution of the incident spatial distribution *I*_opt_ (*x*′) (corresponding to the optical intensity distribution) producing a desired spatiotemporal electric-field distribution *Z̃*_target_ (*x*_o_, ω) at the output. However, one could easily perform waveform synthesis using the experimental measurements of the nonnormalized transfer matrix *T̃*_exp_ (*x*_o_, *x*′, ω) that contains the (generally unknown) incident pulse information (see eq 1). In this approach, the optimization function simply reads as follows
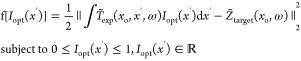
11

**Figure 4:**
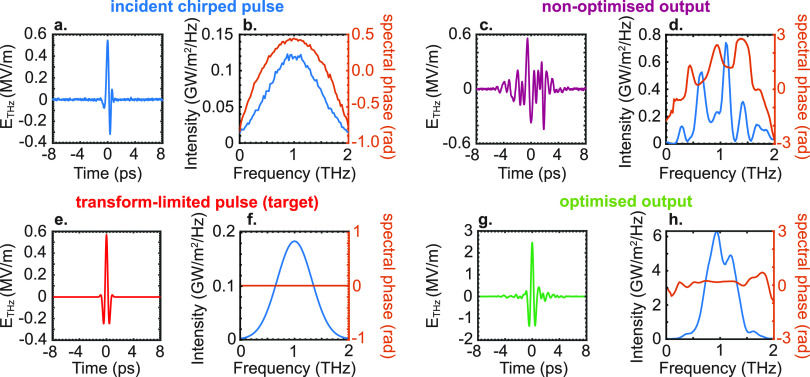
Deterministic coherent control without previous knowledge of the source. (a) Input field-temporal profile corresponding to a chirped pulse. The temporal profile includes the 40 dB noise applied at the detection. (b) Intensity spectral density (blue line) and spectral phase (orange line) for the field profile in panel (a). (c, d) Same as panels (a) and (b) but for a nonoptimized incident pattern. (e, f) Same as panels (a) and (b) for the optimized profile targeted by our optimization routine. (g, h) Same as panels a and b for the optimized output field. The 6.4 × 6.4 mm^2^ sample illumination area is spatially sampled at 200 μm resolution, corresponding to a number of pixels of 32 × 32.

We stress that the optimization routine set by eqs 7 and 11 is field sensitive, and it simultaneously optimizes the amplitude and absolute phase of the transmitted field. This is a radically different scenario from the optical domain, where accessing the absolute phase with arbitrary precision is extremely challenging, and waveform synthesis can be attempted only if the absolute phase profile of the input is known beforehand (e.g., in the case of a transform-limited pulse).^50^ To illustrate this point, in Figure 4, we report the recompression of a chirped THz pulse obtained without any knowledge of the incident field. As shown in Figure 4g,h, the fitness function from eq 11 is well-suited to convert the initially chirped pulse (Figure 4a,b) into a good approximated version of the transform-limited pulse chosen as a target (Figure 4e,f). Analogous to the case in Figure 3a–c, the electric field is measured in the desired focal spot on the output face of the scatterer.

### Time-Resolved Retrieval of Image Object Obscured by a Scattering Medium

The reconstruction of the coherent transmission properties of the scatterer can be directly applied to reconstruct the image of an object concealed by the scattering medium.^62−64^ A particular possibility enabled by our full-field methodology is the possibility of performing phase-sensitive reconstruction, i.e., the reconstruction of samples characterized by complex phase profiles.

**Figure 5:**
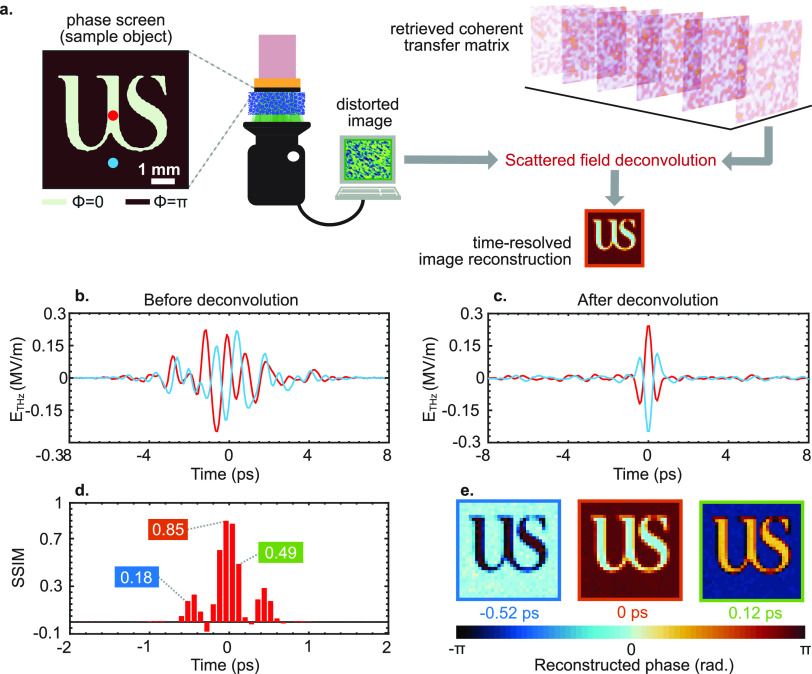
Time-resolved THz phase-sensitive imaging through the scattering medium. (a) Schematic of imaging methodology. Inset: time-resolved reconstruction of the image corresponds to the measurement at *t* = 0 ps in panel (e). (b) Temporal evolution of output speckles corresponding to two different pixels (red and cyan dots shown in panel (a)) before deconvolution. (c) Temporal evolution of reconstructed THz pulse (after deconvolution) for two different pixels (red and cyan dots shown in panel (a)). (d) Structural Similarity (SSIM) index in the time-resolved reconstruction of an image object. (e) Reconstructed phase images at *t* = −0.52, 0, and 0.12 ps. The 6.4 × 6.4 mm^2^ sample illumination area is spatially sampled at 200 μm resolution, corresponding to a number of pixels of 32 × 32 (see Video S1). Logo used with permission from the University of Sussex.

**Figure 6:**
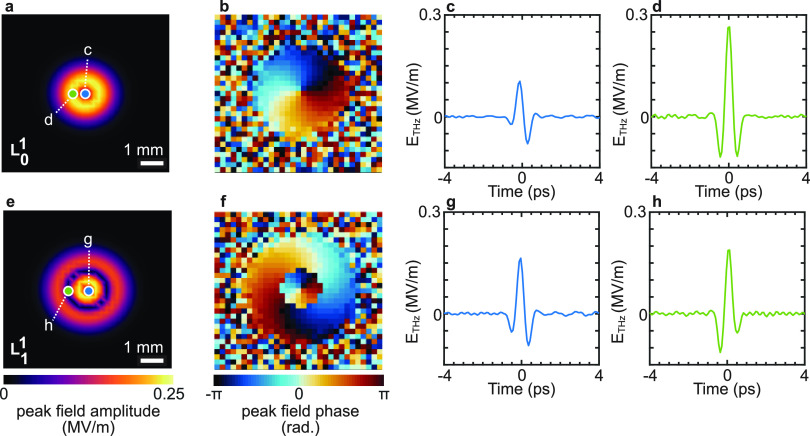
Complex propagation of THz vortex beam through the scattering medium. (a, b) Retrieved spatial field and phase distribution of *L*_0_^1^ vortex beam at *t* = 0 ps. (c, d) Temporal profile of the retrieved THz pulse corresponding to two different pixels for the *L*_0_^1^ vortex beam in panels (a) and (b). (e–h) Same as panels (a–d) for an *L*_1_^1^ vortex beam. The 6.4 × 6.4 mm^2^ sample illumination area is spatially sampled at 200 μm resolution, corresponding to a number of pixels of 32 × 32 (see Video S2).

A numerical implementation of the image reconstruction process is shown in Figure 5a, where we place a phase mask *U*(*x*′) between the generating crystal and the scattering medium. In the frequency domain, the corresponding transmitted field reads as follows
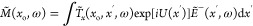
12where *M̃*(*x*_o_, ω) is the time-Fourier transform of space–time measurements. To retrieve the original image from the measurements, we perform a standard deconvolution of the retrieved coherent transfer matrix that yields the time-resolved image *E*_retrieved_ as
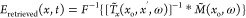
13where (*) denotes a spatial convolution, *F*^–1^ is the inverse time-Fourier transform, and [...]^(−1)^ is the inversion operator. As is customary in deconvolution problems, the main task lies in finding the inverse of (*x*_o_, *x*′, ω). We applied the Moore–Penrose pseudo-inversion method, implemented through a truncated singular-value decomposition.^65^ As shown in Figure 5b, before applying the deconvolution routine, the THz pulses corresponding to the two distinct pixels are thoroughly perturbed, representing the multiplexing of waves due to multiple scattering. Figure 5c shows the waves corresponding to two separate pixels (red and cyan dots) after the deconvolution process. We calculated the Structural Similarity (SSIM) index^66^ to quantify the quality of the reconstruction process, as shown in Figure 5d. SSIM values obtained at *t* = −0.52, 0, and 0.12 ps in our time reference are 0.18, 0.85, and 0.49, respectively, showing high fidelity in the time-resolved reconstruction of the image. The specific reconstruction results shown in Figure 5e are the fixed-time reconstructed phase images of the transmitted field at the same time values. Analogous results for an amplitude-only object (i.e., a metallic mask) are included in Figure S4. As a final example, we extended our image reconstruction approach for the case of THz vortex beam^67^ and simulated the spatiotemporal field-phase profiles for the *L*_0_^1^ and *L*_1_^1^ beam profiles (see Figure S5). We deconvolved the spatial field-phase information of *L*_0_^1^ and *L*_1_^1^ THz vortex beams with the complex scrambled output obtained from propagation through the scattering medium. Figure 6 shows their retrieved spatial field-phase profiles at *t* = 0 ps (Figure 6a,b,e,f). In Figure 6c,d,g,h, we report the temporal profile of the retrieved THz field corresponding to the different pixels marked in Figure 6a,b,e,f.

## Conclusions

In this work, we have theoretically demonstrated a deterministic approach toward the coherent spatiotemporal control of THz waves propagating through a scattering medium. Our methodology combines the nonlinear conversion of optical patterns to THz structured fields with field-sensitive THz field detection, as enabled by state-of-the-art TDS technology. We have shown how the full-wave detection of the scattered THz field enables retrieving the field-sensitive transfer function of the medium directly in a deterministic fashion, as described through a coherent transfer matrix modeling. We sample the complex time-domain elements of the coherent transfer matrix by projecting a sequence of orthogonal Walsh–Hadamard patterns. The TDS allows for a sufficient description of the coherent transfer matrix to enable spatiotemporal control through a direct inversion approach. We identified the spatial profiles that yield a desired output field distribution through a convex constraint optimization routine compatible with real-life experimental conditions. As relevant examples, we demonstrated the formation of single and multiple spatiotemporal foci and the retrieval of complex field distributions and phase-only images concealed by the scatterer. Our results suggest that it is still possible to investigate the scattering unaffected open path via a time-domain deterministic approach in an experimental-driven constrained scenario. Such control could have a profound impact, especially for THz imaging, where wave shaping is generally a challenge. In addition, we envision a role in time-resolved characterization techniques of complex media, including deep-tissue biological imaging.

## Supporting Information Available

The Supporting Information is available free of charge at https://pubs.acs.org/doi/10.1021/acsphotonics.2c00061 The datasets for all figures are freely accessible at: http://doi.org/10.6084/m9.figshare.19447112.

Additional theoretical details, methods, and figures (PDF)
Video for the reconstruction of the phase object in Figure 5 (Video S1) (AVI)
Video for the reconstruction of the THz vortex beams in Figure 6 (Video S2) (MP4)

## Supplementary Material

ph2c00061_si_001.pdf

ph2c00061_si_002.avi

## Abbreviations

THz: terahertz
TDS: time-domain spectroscopy
FROG: frequency-resolved optical gating
SPIDER: spectral phase interferometry for direct electric-field reconstruction
SLM: spatial light modulator
ZnTe: zinc telluride
SNR: signal-to-noise ratio
SSIM: structural similarity index
