# Supplementary material for: Deterministic Terahertz Wave Control in Scattering Media
Source: ACS Photonics. 2022 Jul 19;9(8):2634–42. doi: 10.1021/acsphotonics.2c00061 (PMC9389618; doi:10.1021/acsphotonics.2c00061)
Supplement: Supplementary file 1 — ph2c00061_si_001.pdf [file ph2c00061_si_001.pdf]

Supplementary Information

Deterministic Terahertz wave control in scattering media

Vivek Kumar[1], Vittorio Cecconi[1], Luke Peters[1], Jacopo Bertolotti[2], Alessia Pasquazi[1], Juan Sebastian Totero Gongora, Marco Peccianti[1]*

[1] Emergent Photonics Lab (EP*ic*), Department of Physics and Astronomy, University of Sussex, Brighton, BN1 9QH, U.K.

[2] Department of Physics and Astronomy, University of Exeter, Exeter, Devon EX4 4QL, UK

The Supplementary Information comprises 5 pages and 5 figures.

Supplementary Note 1. Estimation of the spectral correlation bandwidth

We estimated our spectral correlation $\Delta\nu_c$ based on the experimental measurements included in Refs[2–4]. These are seminal works in the study of THz pulse propagation in random media and entail samples composed of closely packed, random collections of Teflon spheres in Teflon cuvettes. Teflon has significantly low absorption in the 0.1-2 THz frequency range of interest to our work, and the spheres have an average radius of $\sim$0.8 mm. In their works, Pearce et al. have reported mean-free-paths of l_s~1-100 mm for samples thickness in the range 1-20 mm and characterized by strong forward scattering (g~1) [2–4]. These quantities correspond to spectral correlation widths in the range from 100 GHz to 500 GHz, as computed with the standard formula $\Delta\nu_c$=2.92$\pi D/L^2$ [,5]. Our particular choice of $\Delta\nu_c$=150 GHz in the revised manuscript was dictated by numerical considerations in the generation of Gaussian-correlated random noise.

Supplementary Note 2. Reconstruction of the coherent transfer matrix

The reconstruction of the coherent transfer matrix can be generally performed by sampling the input-output relation with a series of orthogonal functions. The Walsh-Hadamard basis represents a canonical example known to provide a higher signal to noise (SNR) ratio compared to single-pixel, raster-scan illumination.[1] The general form of the coherent transfer matrix at particular frequency ω is given by:

$$\widetilde{\boldsymbol{T}}(\omega) = \begin{bmatrix} \tilde{T}_{11}(\omega) & \cdots & \tilde{T}_{1N}(\omega) \\ \vdots & \ddots & \vdots \\ \tilde{T}_{N1}(\omega) & \cdots & \tilde{T}_{NN}(\omega) \end{bmatrix} \tag{S1}$$

where $\tilde{T}_{ij}(\omega)$ is the complex-valued field propagator that connects j-th input mode and i-th output pixel and we assume that the transfer matrix is a $N \times N$ square matrix for simplicity. In the presence of broadband illumination, the incident field for a given pattern is expressed as:

$$\tilde{E}_n^-(\omega) = h_p^\pm f(\omega), \tag{S2}$$

where $f(\omega)$ is the THz pulse spectral profile. The spatial profiles $h_p^\pm$ are defined as follows:

$$h_p^\pm = \frac{1}{2} \pm \frac{1}{2} H_p, \tag{S3}$$

where H_P is the p-th column of the $N \times N$ Walsh-Hadamard matrix $\boldsymbol{H}$. With this approach, the corresponding output vectors for each binary pattern $\tilde{c}_p^+(\omega)$ and $\tilde{c}_p^-(\omega)$ can be acquired by performing the Fourier transform of the time-resolved measurements obtained from TDS. In an analogous fashion to differential ghost-imaging approaches, the coefficient corresponding to the p-th Walsh-Hadamard pattern is simply expressed as:

$$\tilde{c}_p(\omega) = \tilde{c}_p^+(\omega) - \tilde{c}_p^-(\omega) \tag{S4}$$

For each frequency, the differential signals are stacked into a measurement matrix $\widetilde{\boldsymbol{M}}(\omega) \in \mathbb{C}^{N\times N}$, and the transfer matrix elements can be obtained by a linear inversion of $\widetilde{\boldsymbol{M}}(\omega)$ for each frequency. In practical terms, the inversion can be obtained in two ways. The simplest approach is to take into account the orthogonality of the Walsh-Hadamard matrix as follows:

$$\widetilde{\boldsymbol{T}}_{reconstructed}(\omega_k) = \frac{1}{f(\omega_k)} \widetilde{\boldsymbol{M}}(\omega_k)\boldsymbol{H} \tag{S5}$$

Due to the presence of noise, however, the inversion is more precisely calculated in a least-square fashion as follows:

$$\widetilde{\boldsymbol{T}}_{reconstructed}(\omega_k) = \mathrm{argmin}_{\boldsymbol{T}\in\mathbb{C}^{N\times N}} \left\| \boldsymbol{T} - \frac{1}{f(\omega_k)} \widetilde{\boldsymbol{M}}(\omega_k)\boldsymbol{H} \right\|^2 \tag{S6}$$

In Figure 2 of the main text we quantitatively estimated the robustness of our approach against additive noise at the detection by computing the Mean Square Error (MSE) of our reconstruction for different values of the detection SNR. The MSE is defined as:

$$MSE(\omega_k) = \frac{1}{N} \left\| \widetilde{\boldsymbol{T}}_{reconstructed}(\omega_k) - \widetilde{\boldsymbol{T}}(\omega_k) \right\|_F^2 = \frac{1}{N} \sum_m \sum_n \left| \widetilde{\boldsymbol{T}}_{reconstructed,mn}(\omega_k) - \widetilde{\boldsymbol{T}}_{mn}(\omega_k) \right|^2 \tag{S7}$$

where $\|\ldots\|_{\mathrm{F}}$ is the Frobenius norm. We compared the MSE values for raster scan and Walsh-Hadamard decomposition (Fig 2 of the main text). As a direct comparison, in Figure S2 we report the same analysis for a single-pixel, raster scan measurement of the transfer matrix elements for different values of the detection SNR.

Supplementary Figures

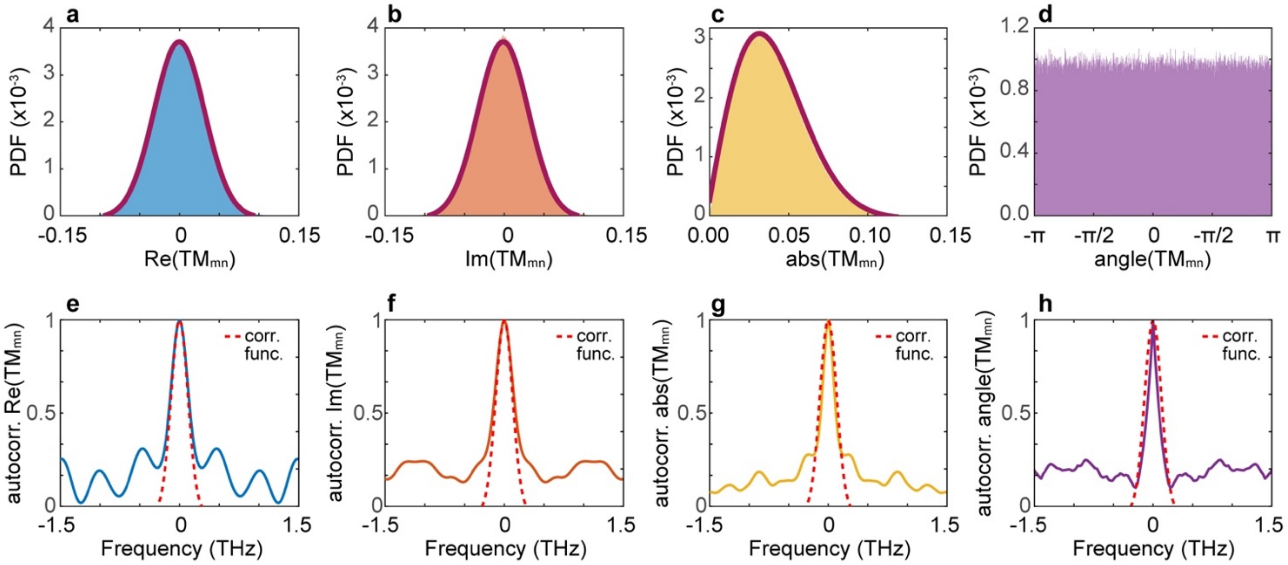

Figure S1: Histograms (Probability density function) and spectral autocorrelation functions for the real part (panels **a,e**), imaginary part (panels **b,f**), amplitude (panels **c,g**), and phase (panels **d,h**) of the transfer matrix elements. The histograms correspond to a single frequency, while the autocorrelation functions are computed for a single pixel. The red lines represent the expected statistical distributions for a circular random Gaussian distribution (Gaussian for the real and imaginary parts, Rayleigh for the amplitudes, and uniform for the phases). In panels **e-h**, the red dotted line is the Gaussian spectral correlation function with a standard deviation of 150 GHz applied to the transfer matrix elements.

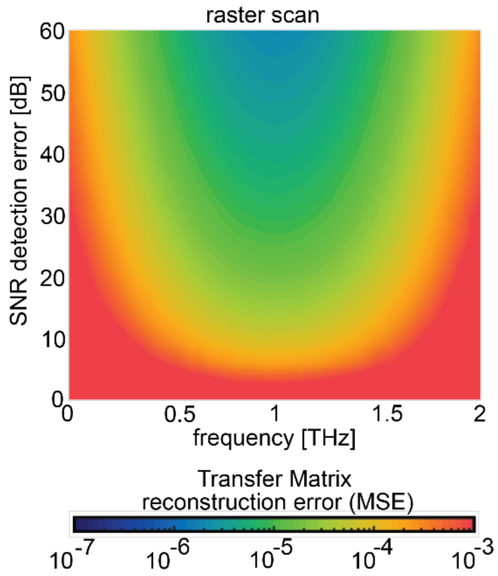

Figure S2: Mean Square Error in the retrieval of coherent transfer matrix using a raster scan reconstruction.

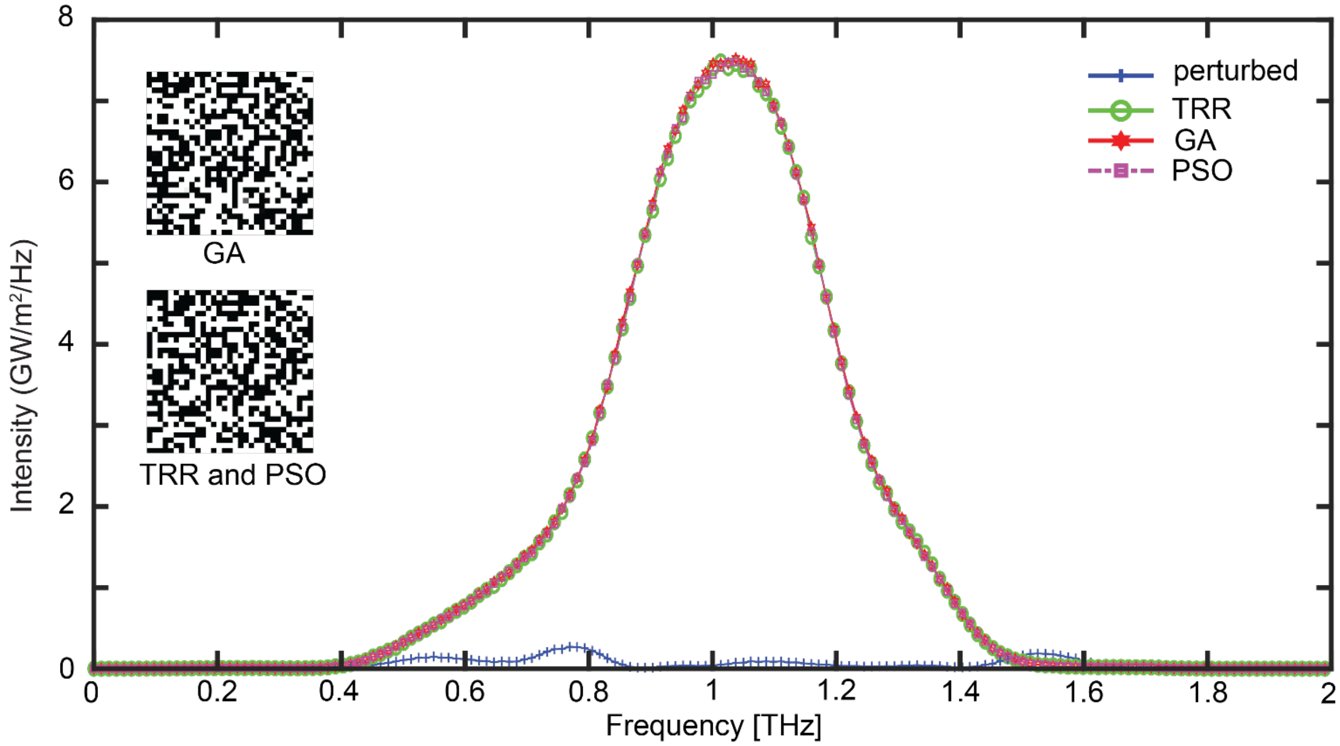

Figure S3. Performance analysis of spatiotemporal focusing of THz waves using Trust-Region-Reflective (TRR), Genetic algorithm (GA) and Particle Swarm Optimisation (PSO) for constraint inversion of the coherent transfer matrix. Optimized intensity spectral density at the focus spot obtained from TRR, GA and PSO (blue: scattered intensity, green circle: using TRR, red star: using GA and pink square: using PSO). Unlike to the GA, TRR and PSO are conversing to the same binary-based pattern solution as shown in the Inset: Optimized binary-based pattern (black: 0, white: 1).

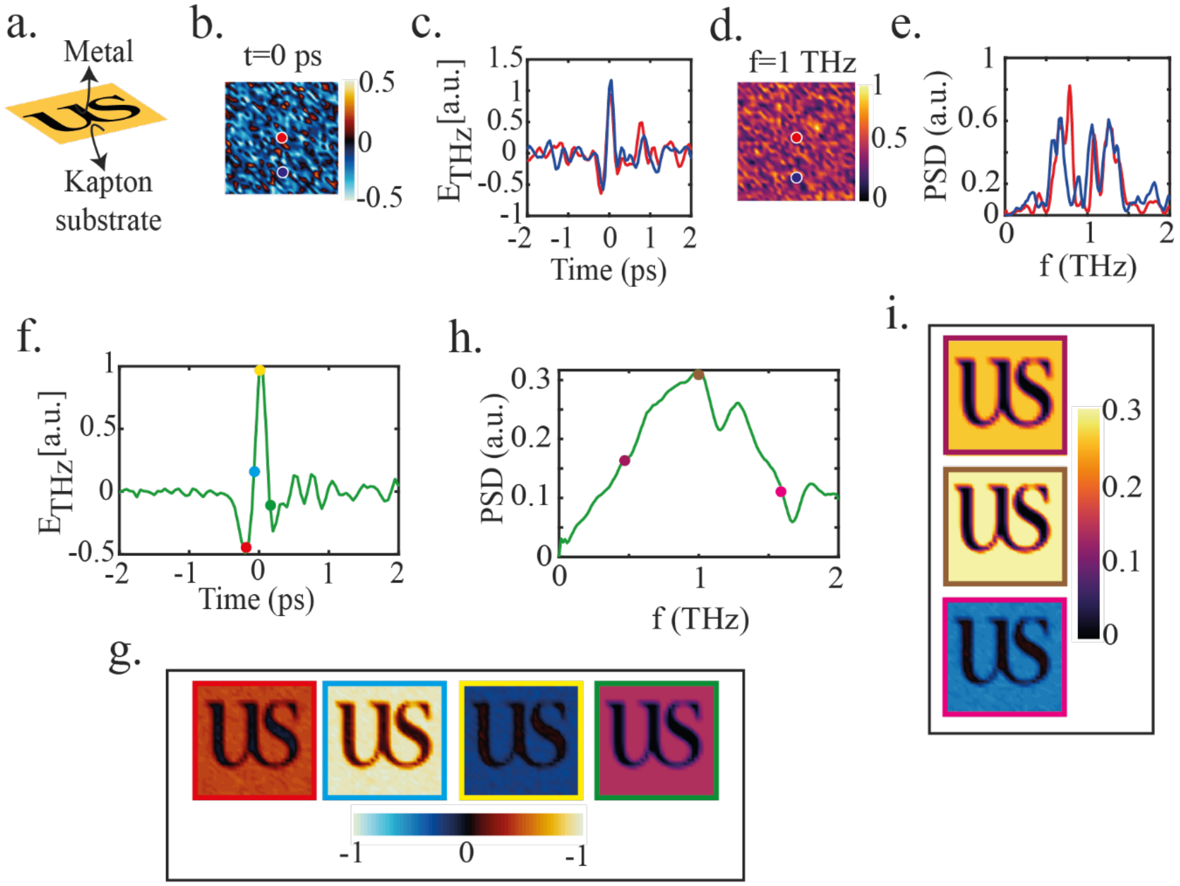

Figure S4: Hyperspectral THz imaging through the scattering medium. **a.** Schematic of metallic image object. **b.** Interference pattern formed at the output after propagation through the scattering medium. **c.** Temporal evolution of output speckle corresponding to two different pixels. **d.** Spectral intensity distribution of scrambled image object propagated through medium at 1 THz. **e.** Intensity profile of output speckle corresponding to two different pixels. **f.** Temporal evolution of reconstructed image object averaged over all the pixels. **g.** Fix time retrieval of images at −0.31 ps, −0.26 ps, 0 ps and 0.17 ps. **h**. The spatially averaged reconstruction of THz spectrum. **i.** Retrieved hyperspectral images at 0.55 THz, 1 THz and 1.55 THz. The 6.4×6.4 mm^2 sample illumination area is spatially sampled at 200 µm resolution. Logo used with permission from the University of Sussex.

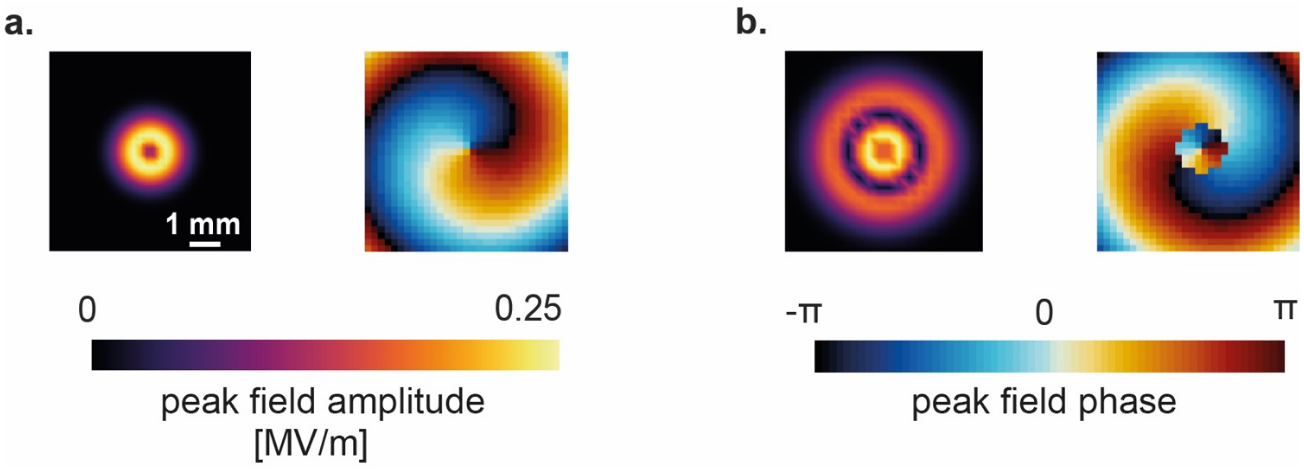

Figure S5: Simulated L_0^1 and L_1^1 THz vortex beams. a. Spatial field and phase distribution for radial index 0 and topological charge 1. **b.** Spatial field and phase profiles for radial index 1 and topological charge 1. THz vortex beams are plotted at the peak of the THz pulse after propagation of 0.5 mm in free space. The 6.4×6.4 mm^2 sample illumination area is spatially sampled at 200 µm resolution.

REFERENCES

(1) Streeter, L.; Burling-Claridge, G. R.; Cree, M. J.; Künnemeyer, R. Optical Full Hadamard Matrix Multiplexing and Noise Effects. *Applied optics* **2009**, *48* (11), 2078–2085.

(2) Pearce, J.; Mittleman, D. M. Propagation of Single-Cycle Terahertz Pulses in Random Media. *Opt. Lett., OL* **2001**, *26* (24), 2002–2004. https://doi.org/10.1364/OL.26.002002.

(3) Pearce, J.; Jian, Z.; Mittleman, D. M. Statistics of Multiply Scattered Broadband Terahertz Pulses. *Phys. Rev. Lett.* **2003**, *91* (4), 043903. https://doi.org/10.1103/PhysRevLett.91.043903.

(4) Pearce, J. Multiple Scattering of Broadband Terahertz Pulses. PhD Thesis, Rice University, 2005.

(5) Aulbach, J.; Gjonaj, B.; Johnson, P. M.; Mosk, A. P.; Lagendijk, A. Control of Light Transmission through Opaque Scattering Media in Space and Time. *Phys. Rev. Lett.* **2011**, *106* (10), 103901. https://doi.org/10.1103/PhysRevLett.106.103901.
